# Identification of haplotype tag single nucleotide polymorphisms within the receptor for advanced glycation end products gene and their clinical relevance in patients with major trauma

**DOI:** 10.1186/cc11436

**Published:** 2012-07-24

**Authors:** Ling Zeng, An-qiang Zhang, Wei Gu, Jian Zhou, Lian-yang Zhang, Ding-yuan Du, Mao Zhang, Hai-yan Wang, Jun Yan, Ce Yang, Jian-xin Jiang

**Affiliations:** 1 State Key Laboratory of Trauma, Burns and Combined Injury, Institute of Surgery Research, Daping Hospital, Third Military Medical University, Changjiang Road 10, Yuzhong District, Chongqing, 400042, China; 2 Department of Traumatic Surgery, Daping Hospital, Third Military Medical University, Changjiang Road 10, Yuzhong District, Chongqing, 400042, China; 3 Chongqing Emergency Medical Center, Jiankang Road, Yuzhong District, Chongqing, 400042, China; 4 Department of Emergency Medical Center, the Second Affiliated Hospital, Zhejiang University, Jiefang Road 88, Zhejiang, 310009, China

## Abstract

**Introduction:**

The receptor for advanced glycation end products (*RAGE*) has been considered as one of the major pattern recognition receptors and plays an important role in the development of sepsis and multiple organ dysfunction in critical illnesses. Although genetic variants of the *RAGE *gene have been shown to be well associated with susceptibility to some inflammatory diseases, little is known about their clinical relevance in the development of sepsis in critical ill patients.

**Methods:**

Four genetic variants were selected from the entire *RAGE *gene and genotyped using pyrosequencing and polymerase chain reaction-length polymorphism methods. Association studies were performed in two independent Chinese Han populations.

**Results:**

Among the four genetic variants, only the rs1800625 polymorphism was significantly associated with sepsis morbidity rate and multiple organ dysfunction (MOD) scores in patients with major trauma both in Chongqing (n = 496) and Zhejiang (n = 232) districts, respectively. Results from *ex vivo *responsiveness of peripheral blood leukocytes indicated that the rs1800625 polymorphism was well associated with decreased production of TNFα. In addition, the rs1800625 polymorphism could significantly inhibit the promoter activities of the *RAGE *gene.

**Conclusions:**

The rs1800625 polymorphism is a functional variant, which might be used as a relevant risk estimate for the development of sepsis and multiple organ dysfunction syndrome in patients with major trauma.

## Introduction

The receptor for advanced glycation end products (*RAGE*), not only limiting to mediation of advanced glycation end products (AGEs), has been recognized as a multiligand receptor, especially playing pivotal roles in innate immune responses as a pattern-recognition receptor (PRR) in sensing both pathogen-associated molecular patterns (PAMPs) and endogenous damage-associated molecular patterns (DAMPs) [1-6]. *RAGE *could up-regulate the capacity of leukocytes to kill bacteria [2] and facilitates host defense during bacterial infection [3,4]. Besides its anti-microbial effects, *RAGE *also acts as an endothelial adhesion receptor for leukocyte integrins and promotes leukocyte recruitment [5]. Engagement of *RAGE *by its diverse ligands, such as lipopolysaccharides, high-mobility group box 1 protein, AGEs, results in receptor-dependent signaling and amplification of pro-inflammatory responses [1]. *RAGE *is up-regulated at levels of both mRNA and protein in sepsis [6]. Administration of AGE-modified protein has the potential to activate *RAGE*/NF-kB-mediated inflammatory reactions, causing increased mortality in experimental peritonitis [7]. In addition, the genomic deletion of *RAGE *[8,9], or inhibition of *RAGE *signaling [8,10] results in markedly decreased septic response and significantly increased survival rates in a mouse model of sepsis. Therefore, *RAGE *has been considered to be one of the major PRRs responsible for the development of sepsis [11] and its subsequent organ dysfunction [12-14]. It might be used as a potential molecular marker and therapeutic target for sepsis [15-17].

The *RAGE *gene is located on chromosome 6p21.3 in the major histocompatibility complex (MHC) locus in the class III region [18]. Growing evidence indicates that allelic variation within the key domains of *RAGE *gene may influence the magnitude of proinflammatory response, thereby affecting susceptibility to acute and chronic inflammatory diseases [19]. A total of 97 genetic variants have been identified so far in the entire *RAGE *gene. However, only three of them (rs1800625, rs1800624 and rs2070600) have been well studied in relation to their clinical relevance. The rs1800625 is revealed in association with increased risk for diabetes [20], diabetic retinopathy [21] and colorectal cancer [22]. The rs1800624 polymorphism was shown to be associated with increased susceptibility to Crohn's disease [23], coronary artery disease [24], in-stent restenosis after coronary stent implantation [25], ischemic heart disease [26], and myocardial infarction [27]. The rs2070600 polymorphism revealed increased risk of rheumatoid arthritis [28], multiple sclerosis [29] and coronary artery disease in non-diabetics [30]. In spite of the above findings, there also have been plenty of controversial reports with respect to the clinical relevance of the above three gene polymorphisms [26,27,29,31-36]. In addition, other polymorphisms, such as 2184A/G, 2245G/A, rs17493811 and rs9469089, also have been reported in relation to diabetes [37-39]. However, little is known about the clinical relevance of the genetic variants of *RAGE *gene in relation to the development of sepsis and multiple organ dysfunction syndrome (MODS).

To comprehensively assess the clinical relevance of the common genetic variants within the entire *RAGE *gene, haplotype bins were constructed to identify haplotype tagging single nucleotide polymorphisms (SNPs) (htSNPs) within the entire *RAGE *gene and its surrounding regions. In view of that, the majority of trauma patients are young people with little history of pre-existing diseases [40]. The trauma patient cohort is appropriate to investigate the association of genetic factors with susceptibility or resistance to sepsis in critically ill patients. Four genetic variants were selected, and two independent Chinese Han populations were recruited in this study cohort. The rs1800625 polymorphism (-429T/C) was demonstrated to be clinically relevant and have functional activity.

## Materials and methods

### Study population and clinical evaluation

A total of 728 unrelated patients with major trauma recruited in this study were Han Chinese and from Chongqing in south-western China (n = 496) and Zhejiang in eastern China (n = 232), respectively. The trauma patients were admitted to the Department of Trauma Surgery in the Daping Hospital and the Chongqing Emergency Medical Center between 1 January 2005 and 1 May 2011, and to the Department of Trauma and Emergency in the Second Affiliated Hospital, Zhejiang University between 1 January 2008 and 1 May 2011. They were enrolled in the study if they met the following inclusion criteria: 1) were between 18 and 65 years of age, 2) expected an Injury Severity Score (ISS) greater than 16 combined with the presence of at least one life threatening injury and at least one additional severe injury in another part of the body, and 3) had a probability of survival greater than 48 h. Patients were not eligible if they had penetrating injuries or preexisting cardiovascular, respiratory, renal, hepatic, hematologic or immunologic diseases. ISS was performed according to the abbreviated injury scale by independent evaluators [41]. Standard demographic, laboratory and clinical data were extracted from a prospectively collected database. The protocol was approved by the Ethical and Protocol Review Committee of the Third Military Medical University, and informed consent was obtained from the patients and the patients' next of kin. Patient confidentiality was preserved according to the guidelines for studies of human subjects.

The patients with major trauma were prospectively monitored after admission by physicians who did not know the genotypes. The definitions of sepsis and infection were shown in Additional file 1. Daily physiologic and laboratory data were collected during the ICU stay and clinical events were recorded thereafter, until death or hospital discharge. Multiple organ dysfunction scores were calculated as the sum of the simultaneously obtained individual organ scores on each hospital day [42]. Neurological scoring was not performed because every patient was sedated. MODS is defined as a Marshall score of 4 or more for at least two consecutive days based on the comparative studies reported by A Sauaia [43].

### Tag SNP selection

The human *RAGE *gene (Accession Number: NC_000006) was pinpointed to chromosome 6, position 32148745-32152022 (data retrieved from Genbank in the website of NCBI). The *RAGE *5' flanking region from -505 in a 5'direction has been shown to overlap with *PBX2*, a gene that has a pseudogene copy on chromosome 3, which might make any studies of polymorphisms in this duplicated region potentially fraught with error [44]. Therefore, besides all exons and introns of the *RAGE *gene, 505-bp upstream of the transcription start site and 5,000-bp downstream of the stop codon were included (8.872 kb total).

**Figure 1:**
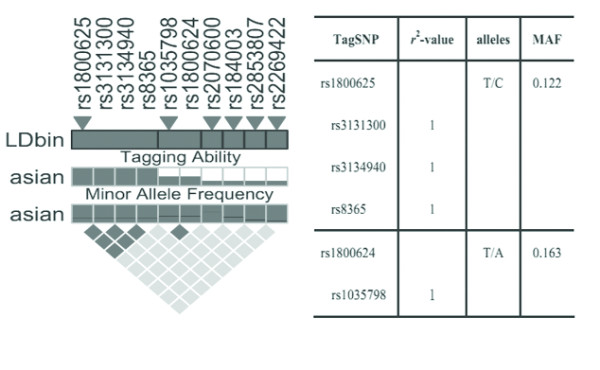
**Overview of selected tSNPs within the entire *RAGE *gene**. (**A**) The pair-wise analysis of linkage disequilibrium (LD), based on *r*^2^, among the 10 SNPs with a minor allele frequency ³5% within the *RAGE *gene and 505 bp up- and 5,000 bp down-stream regions. The selected tSNPs are indicated by trigones. A LD-plot of the 10 SNPs is displayed by using *r*^2^-black and white color scheme. Black represents very high LD (*r*^2 ^= 0.8-1), and white indicates the absence of correlation (*r*^2 ^= 0-0.2) between SNPs. (**B**) The two tSNPs and SNPs that are indirectly measured by them are listed with corresponding *r*^2-^values. Major and minor alleles of the selected tSNPs are given with their frequencies, based on the HapMap data for the CHB population.

Genetic variation data for the entire *RAGE *gene and its surrounding selected regions were obtained from the healthy Chinese Han Beijing (CHB) population of HapMap. From this database, a total of 11 SNPs have been identified in the CHB population (Table 1). Ten of them were common SNPs with a minor allele frequency (MAF) more than or equal to 0.05, which were then selected for the analysis of htSNPs (Figure 1). Haplotype blocks (bins) were constructed using LDselect (Christopher S. Carlson and Deborah A. Nickerson, Seattle, Washington, USA), a software package that provides computation of linkage disequilibrium (LD) statistics and population haplotype patterns from genotype data [45]. This algorithm selects a subset of htSNPs that efficiently describe all common patterns of variants in a gene, based on two primary criteria: 1) the MAF is more than or equal to 0.05 and 2) the minimum level of association between assayed and unassayed SNPs, measured by the linkage disequilibrium statistic *r*^2 ^is more than or equal to 0.8. Given these parameters, LDSelect selects a subset of htSNPs that captures all known common genetic variants within the entire gene.

**Table 1:** SNPs identified within whole *RAGE *gene

**NO**.	rs number	Location	Variation	MAF	Region
1	rs1800625	-429	T/C	0.122	promoter
2	rs1800624	-374	T/A	0.163	promoter
3	rs3131300	79	A/G	0.122	Intron1
4	rs2070600	570	G/A	0.289	Exon3
5	rs2269422	719	A/G	0.057	Intron3
6	rs1035798	791	C/T	0.167	Intron3
7	rs184003	1717	G/T	0.116	Intron5
8	rs3134940	2197	A/G	0.11	Intron7
9	rs2853807	2441	C/T	0.068	Intron7
10	rs2071288	2753	G/A	0.022	Intron8
11	rs8365	3610	C/G	0.114	3'-flanking

Genetic variation data for the entire *RAGE *gene was obtained from the HapMap project for 45 members of the Chinese Han Beijing (CHB) population. MAF, minor allele frequency; SNPs, single nucleotide polymorphisms

To determine the possible functionality of the htSNPs selected from the 5'-flanking region of the *RAGE *gene, on-line software was used to analyze the effect of these SNPs on potential transcription factor binding sites.

### Genotyping

Blood specimens were collected in tripotassium ethylenediamine tetraacetic acid sterile tubes from trauma patients immediately after admission to avoid the effect of blood transfusion. The genomic DNA was isolated from whole blood using the Wizard genomic DNA purification kit (Promega, Madison, WI, USA) according to the manufacturer's protocol. Pyrosequencing was used for genotyping of rs1800625, rs1800624 and rs2070600 polymorphisms according to our previous reports [46]. Polymerase chain reaction (PCR) - length polymorphism (LP) approach was used for genotyping of the 63 bp ins/del variant. The PCR primers and the annealing temperature were shown in Additional file 2, Table S1. Genotyping was performed in a blinded fashion without knowledge of the patients' clinical data, and approximately 10% of the samples were genotyped in duplicate to monitor genotyping quality.

### *Ex vivo *tumor necrosis factorα production

A human whole-blood assay was used as in our previous method. In brief, aliquots of whole blood collected from the trauma patients immediately after admission were mixed 1:1 with Roswell Park Memorial Institute (RPMI) 1640 culture medium (Thermo Scientific, Beijing, China), and incubated with 100 ng/ml LPS (*Escherichia coli *O26:B6, Difco Laboratories, Detroit, MI, USA) in a sample mixer at 37°C for 4 h. After centrifugation, the supernatants were aspirated and aliquoted for sto*RAGE *at -80°C. Tumor necrosis factor-alpha (TNF-α) in the supernatants was assayed with a sandwich enzyme linked immunosorbent assay (ELISA), according to the manufacturer's instructions (Endogen, Woburn, MA, USA). The detection limits of the assay were 4 pg/ml.

### Functionality of the rs1800625 polymorphism

The possible effect of rs1800625, which is located in the 5'-flanking region (-429T/C), on the promoter activity was investigated using a reporter gene assay system [47]. A 531-bp sequence (-504 approximately +26) of the *RAGE *gene containing T or C allele at position -429 were obtained according to the method described in Additional file 3 and were directly inserted into a promoterless pGL3-Basic vector (Promega, Madison, WI, USA) containing the firefly luciferase gene as a reporter. Human U937 were cultured in RPMI 1640 medium (Hyclone, Logan, UT, USA) containing 10% fetal calf serum, 3 mM glutamine, penicillin-streptomycin (100 U/mL for each), and 23 mM sodium bicarbonate at 37°C in a humidified 5% CO_2 _air atmosphere. After incubation for 24 hours, the cultured cells were co-transfected with 0.8 μg of the constructed vectors or pGL3-Basic original plasmid and 20 ng control Renilla luciferase reporter plasmid pRL-CMV using Lipofectmine 2000 system (Invitrogen, Carlsbad, CA, USA). At 24 hours post-transfection, the cells were treated with LPS (100 ng/mL) for 24 hours. Then luciferase activity of the transfected cells was measured using the Luciferase Assay System (Promega) following the supplier's protocol on a Luminoskan Ascent luminometer (Thermo Labsystems, Helsinki, Finland). Transfection efficiency was normalized by measuring the luciferase activity of control plasmid pRL-CMV. Luminescence experiments were performed in triplicate with each transfection. Three independent transfections were performed for each constructed vector. Results are expressed as fold increase in relative luciferase activity of the *RAGE *promoter construct vectors compared with the relative luciferase activity of pGL3-Basic.

### Statistical analysis

Sample size was calculated using online Power and Sample Size Program software (William D Dupont and Walton D Plummer, Nashville, Tennessee, USA) [48]. The desired power of our study was set at 80% with a significance level of 0.05 in a two-sided test. We chose the log-additive inheritance model, which is the most suitable for polygenic diseases.

Allele frequencies for each SNP were determined by gene counting. Genotype distribution of each SNP was tested for departure from Hardy-Weinberg equilibrium (HWE) using χ^2 ^analyses. The extent of pair-wise linkage disequilibrium between polymorphisms was determined by the Haploview (version 4.0) software (Jeffrey C Barrett and Mark J Daly, Cambridge, MA, USA). The association between polymorphisms and supernatant TNFα was determined using one-way analysis of variance. The association between polymorphisms and MOD scores was performed using analysis of one-way ANOVA testing with age, sex ratio and injury severity to adjust for possible confounding effects. Three genetic models (dominant, recessive and allele-dose effects) were used. The association of genotypes with sepsis morbidity rate was determined by χ^2 ^analysis. Odds ratios with 95% confidence intervals were calculated by multiple logistic regression analyses to estimate the relative risk of sepsis. Age, sex ratio and injury severity were used as covariances of multiple logistic regression. *P-*values smaller than 0.05 after Bonferroni correction for multiple testing were considered significant. All statistic analysis was carried out using SPSS Version 13.0.

## Results

### Construction of haplotype bins and selection of htSNPs

There are a total of 10 SNPs with a minor allele frequency of more than or equal to 5% in the CHB population, which constructed two haplotype bins (Figure 1). Based on the analysis of tagging threshold of *r*^2 ^of SNPs in each bin, one htSNP was selected from each bin for genotyping. In combination with previous association studies [21], the rs1800625 and rs1800624 polymorphisms were selected as htSNP of the respective bins (Figure 1). The rs2070600 polymorphism, though not forming any bin with other SNPs, was still selected due to its nonsynonymous variation, showing a substitution of the conserved glycine to serine at amino acid 82 (Gly82Ser) [19]. In addition, a deletion variant (63 bp ins/del) was identified in the region of 5'-flanking region (-407 to -345). This variant, although not being included in the current public gene polymorphism database, was shown to affect the promoter activities of the *RAGE *gene [21]. As a result, this variant was also selected in this study cohort.

### Overall clinical characteristics of patients with major trauma

There were two independent patient cohorts, consisting of 496 and 232 individuals, respectively. All patients survived at least 48 hours after admission and completed genotyping. Baseline data of the patients are shown in Table 2. Patients were severely injured and mostly young. Sepsis morbidity rate was 40.7% and 37.9%, respectively in the Chongqing and Zhejiang cohorts. No pathogens were identified in blood cultures in 53.0% and 50.0% of the patients in both cohorts as the causative microorganism for sepsis, although they had an identified site of infection. The common pathogens identified in this study cohort were *Staphylococcus aureus*, coagulase-negative staphylococci, *Klebsiella pneumoniae, Acinetobacter baumannii, Pseudomonas aeruginosa, Escherichia coli, Enterococcus *spp. and *Enterobacter cloacae*. Gram negative infection accounted for about 21% and 24.6%, Gram positive infection for about 18% and 15.9%, and mixed infection for about 5.8% and 7.3%, respectively, in the Chongqing and Zhejiang cohorts. Median time point for sepsis occurrence in the whole study cohort was 6.0 days (interquartile range 5.0 to 9.0 days. Chongqing: median 7.0 days, interquartile range 5.0 to 8.0 days; Zhejiang: median 6.0 days, interquartile range 5.0 to 7.0 days). Organ dysfunction occurred in 72.1% and 73.2% of the patients in both cohorts, among whom 213 (42.9%) and 96 (41.4%), respectively, had two or more organ dysfunctions. Among the patients with MODS, those with sepsis accounted for 40.7% and 37.9% in Chongqing and Zhejiang populations, respectively. Among the patients with sepsis, the median time point for MODS occurrence was shown to be 8.0 days (interquartile range 6.5 to 10.5 days) in Chongqing, and 9.0 days (interquartile range 7.0 to 11.0 days) in Zhejiang patients, respectively. With respect to the patients without sepsis, the median time point for MODS occurrence was 5.0 days (interquartile range 4.0 to 8.0 days) in Chongqing, and 5.5 days (interquartile range 3.5 to 7.5 days) in Zhejiang patients, respectively.

**Table 2:** Overall clinical characteristics of patients with major trauma

	Chongqing (N = 496)	Zhejiang (N = 232)
Age (yrs)	40.4 ± 14.2 (18 to 65)	41.0 ± 13.1 (18 to 65)
Male/female, n	395/101	190/42
Injured body regions, n (%)		
Head, n	187 (37.7)	73 (31.5)
Thorax, n	205 (41.3)	142 (61.2)
Abdomen, n	136 (27.4)	74 (31.9)
Extremities, n	252 (50.8)	165 (71.1)
Number of regions injured, n (%)		
One, n	145 (29.2)	74(31.9)
Two, n	133 (26.8)	59 (25.4)
Three or above, n	80 (16.1)	37 (15.9)
ISS	22.0 ± 10.5	23.3 ± 8.7
≥16, <25, n (%)	301 (60.7)	131 (56.5)
≥25, n (%)	195 (39.3)	101 (43.5)
Organ dysfunction, n (%)		
One, n	132 (26.6)	76 (32.8)
Two, n	125 (25.2)	59 (25.4)
Three or above, n	72 (14.5)	35 (15.1)
Sepsis, n (%)	202 (40.7)	88 (37.9)
Source of infection, %		
Respiratory tract infection	41.6	39.8
Primary bloodstream infection	21.8	23.9
Urinary tract infection	16.3	11.4
Catheter associated infection	10.9	9.1
Wound infection	7.4	9.1
Others*	2.0	6.8
Pathogens, % (positive blood cultures)		
Gram-negative	21.0	24.6
Gram-positive	18.0	15.9
Fungi	2.2	2.2
Mixed Gram negative and positive	5.8	7.3
Negative blood cultures	53.0	50.0

### Allele frequencies and genotype distribution

The MAF of the rs2070600, rs1800624 and rs1800625 polymorphisms among the trauma patients were similar to those among the CHB population in the HapMap database (Table 3). The MAF of the 63 bp ins/del variant was 7.7% and 6.3% in the Chongqing and Zhejiang districts, respectively. This seems to be higher in the Chinese Han population when compared with that in Western populations, among whom the MAF of the 63 bp ins/del variant is less than 1% [21]. The genotype distribution of the four genetic variants was in agreement with the Hardy-Weinberg equilibrium (*P *>0.05, Table 3), indicating that both allele and genotype frequencies of these variants in the population remain constant; that is, they are in equilibrium from generation to generation.

**Table 3:** Distribution of the four variants in the *RAGE *gene among trauma patients

			MA (%)		Genotypes, n (%)			HWE
		
	SNPs	N	Patients	Databank	wild	Heterozygous	variant	*P-*values
**Chongqing**	63bp ins/del	496	7.7		423 (85.3)	70 (14.1)	3 (0.6)	0.96
	rs2070600	496	29.4	28.9	247 (49.8)	206 (41.5)	43 (8.7)	0.99
	rs1800624	496	14.8	16.3	364 (73.4)	117 (23.6)	15 (3.0)	0.14
	rs1800625	496	14.7	12.2	365 (73.6)	116 (23.4)	15 (3.0)	0.128

**Zhejiang**	63bp ins/del	232	6.3		203 (87.5)	29 (12.5)	0 (0)	0.31
	rs2070600	232	27.4	28.9	126 (54.3)	85 (36.6)	21 (9.1)	0.23
	rs1800624	232	11.6	16.3	181 (78.0)	48 (20.7)	3 (1.3)	0.93
	rs1800625	232	15.9	12.2	163 (70.3)	64 (27.6)	5 (2.2)	0.66

### Clinical relevance of the four genetic variants in trauma patients in the Chongqing district

We first selected 496 Chinese Han patients with major trauma in the Chongqing district to investigate the clinical relevance of the four variants (63 bp ins/del, rs2070600, rs1800624 and rs1800625) of the *RAGE *gene. As shown in Table 4, there were no significant differences in age, gender ratio and ISS among patients stratified according to the different genotypes of each variant. Among the four genetic variants selected in this study, only the rs1800625 was shown to be significantly associated with the risk for development of sepsis and MODS in major trauma patients. The patients carrying the variant C allele revealed a significantly lower sepsis morbidity rate and MOD scores, when compared with those carrying the T allele (*P *= 0.003 for sepsis morbidity rate and *P *= 0.015 for MOD scores in case of dominant effect, *P *= 0.032 for MOD scores in case of recessive effect). Data from regression analyses further indicated that the association of this polymorphism was in significant allele-dose effect with sepsis morbidity rate (OR = 0.428, 95% CI: 0.276 to 0.886, *P *= 0.026), and MOD scores (*P *= 0.045), respectively. There were no significant differences in the sepsis morbidity rate and MOD scores among different groups when patients were stratified according to the genotypes of the other three genetic variants.

**Table 4:** Clinical relevance of four variants among trauma patients

	Polymorphic sites	Genotypes	N	Age (yr)	Sex (M/F)	ISS	Sepsis, n/%	MOD score
**Chongqing**		ins/ins	423	40.2 ± 14.6	334/89	22.3 ± 10.5	178 (42.1)	6.4 ± 2.4
	63 bp ins/del	ins/del	70	40.9 ± 11.1	59/11	20.5 ± 10.5	23 (32.9)	6.1 ± 2.8
		del/del	3	47.3 ± 18.8	2/1	24.3 ± 8.5	1 (33.3)	6.0 ± 1.7
	
		GG	247	39.5 ± 14.4	198/49	22.2 ± 11.4	100 (40.5)	6.7 ± 2.6
	rs2070600	GA	206	41.0 ± 14.4	161/45	21.9 ± 9.4	84 (40.8)	6.1 ± 2.3
		AA	43	42.5 ± 11.5	36/7	22.0 ± 10.4	18 (41.9)	5.7 ± 1.6
	
		TT	364	40.4 ± 14.3	291/73	22.2 ± 10.4	148 (40.7)	6.3 ± 2.4
	rs1800624	TA	117	40.5 ± 14.3	91/26	21.7 ± 11.0	51 (43.6)	6.5 ± 2.5
		AA	15	39.7 ± 12.0	13/2	20.2 ± 8.8	3 (20.0)	7.0 ± 2.6
	
		TT	365	40.0 ± 14.1	291/74	22.4 ± 10.5	163 (44.7)	6.9 ± 2.4
	rs1800625	TC	116	42.4 ± 14.4	89/27	21.3 ± 10.7	35 (30.2)	6.0 ± 2.2
		CC	15	37.3 ± 9.9	15/0	17.2 ± 9.8	4 (26.7)	5.3 ± 1.5
							a1	a2, b1, c1

**Zhejiang**		ins/ins	203	41.0 ± 12.9	165/38	23.3 ± 8.6	74 (36.4)	6.9 ± 3.4
	63bp ins/del	ins/del	29	41.1 ± 14.9	25/4	22.7 ± 9.3	14 (48.3)	8.4 ± 3.0
		del/del	0	-	-	-	0 (0)	-
	
		GG	126	41.3 ± 13.4	102/24	22.7 ± 8.6	48 (38.1)	7.1 ± 3.1
	rs2070600	GA	85	40.7 ± 12.4	70/15	23.0 ± 8.5	35 (41.2)	6.9 ± 3.6
		AA	21	40.6 ± 14.5	18/3	27.5 ± 8.6	5 (23.8)	6.9 ± 2.8
	
	rs1800624	TT	181	41.0 ± 13.3	150/31	23.4 ± 8.7	70 (38.7.)	7.0 ± 3.5
		TA	48	40.6 ± 13.0	38/10	22.4 ± 8.3	16 (33.3)	7.4 ± 3.2
		AA	3	44.7 ± 7.1	2/1	28.0 ± 14.1	2 (67.7)	6.7 ± 2.3
	
		TT	163	41.4 ± 13.2	138/25	23.7 ± 8.5	70 (42.9)	7.3 ± 3.3
	rs1800625	TC	64	40.9 ± 13.2	47/17	21.8 ± 8.6	16 (25.0)	6.9 ± 3.2
		CC	5	37.7 ± 15.7	4/1	24.3 ± 12.0	2 (40.0)	6.3 ± 2.5
							a3	a4, c2

A, dominant effect (variant homozygotes +heterozygotes vs. wild homozygotes) as analyzed by ANOVA, ^a1^*P *= 0.003, ^a2^*P *= 0.015, ^a3^*P *= 0.016, ^a4^*P *= 0.042; b, recessive effect (variant homozygotes vs. heterozygotes + wild homozygotes); as analyzed by ANOVA, ^b1^*P *= 0.032; c, Allele Dose Effect as analyzed by linear regression, ^c1^*P *= 0.045, ^c2^*P *= 0.048.

### The clinical relevance of the four variants in trauma patients in the Zhejiang district

In order to confirm the biological significance of the four genetic variants selected in this study, we further investigated their clinical relevance in another Chinese Han population of trauma patients, who lived in the Zhejiang province of eastern China. As expected, only rs1800625 was shown to be significantly associated with risk of sepsis and MODS in trauma patients in the Zhejiang district. The sepsis morbidity rates and MOD scores were significantly lower in the patients carrying the variant C allele compared with those carrying the T allele (*P *= 0.016 and *P *= 0.042 for sepsis morbidity rates and MOD scores in case of dominant effect, Table 4). Data from linear regression analysis revealed that the association of this polymorphism was in borderline significant allele-dose effect with MOD scores (*P *= 0.048).

### Effect of rs1800625 on LPS-induced TNF-α production

**Figure 2:**
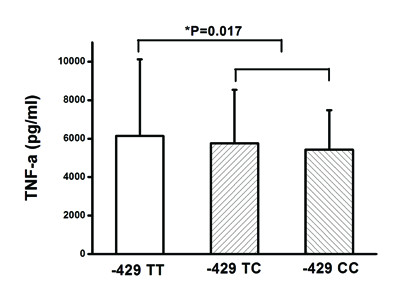
**Effect of the rs1800625 (-429T/C) on LPS-induced TNF-α production**. One-way ANOVA was used to assess statistical significance. *P = 0.017 for dominant association (TT + TC vs. TT).

As shown in Figure 2, the rs1800625 polymorphism was well associated with the LPS responsiveness of peripheral blood leukocytes. LPS-induced TNF-α production was significantly lower in patients with the variant C allele than that in those with wild T allele (CC genotype: 5423.4 ± 2059.4 pg/ml, TC genotype: 5763.1 ± 2771.8 pg/ml and TT genotype: 6146.7 ± 3978.4 pg/ml, *P *= 0.017 for dominant effect).

### Effect of the rs1800625 on promoter activity

**Figure 3:**
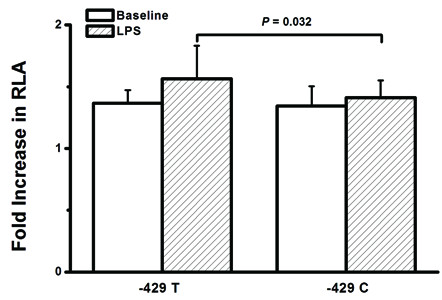
**Effect of the -429T/C polymorphism on the transcription activity of the *RAGE *promoter**. Relative luciferase activity (RLA) was measured in U937 cells transfected with -429T or -429C plasmid constructs as described in methods. The fold increase of RLA was significantly lower in cells transfected with variant C allele than those transfected with wild T allele (*P *= 0.032).

In view of the location of the rs1800625 in the 5'-flanking region of the *RAGE *gene, we further hypothesized that the T→C variation of this polymorphism might affect the promoter activities of the *RAGE *gene. Figure 3 showed that the fold increase of relative luciferase activity (RLA) was significantly lower in cells transfected with variant C allele than those transfected with wild T allele (1.36 ± 0.11 vs. 1.57 ± 0.27, *P *= 0.032).

## Discussion

It has been demonstrated that inappropriate immune inflammatory response contributes to the development of sepsis and MODS in critically ill patients [40]. Increasing evidence suggests that genetic variants, particularly single nucleotide polymorphisms (SNPs), are critical determinants for inter-individual differences in both inflammatory responses and clinical outcome [48,49]. Delineating the variation in genes and associated differences in immune inflammatory response might contribute to the development of new genetically tailored diagnostic and therapeutic interventions that will improve outcome in critically ill patients. *RAGE*, a member of the immunoglobulin superfamily of cell surface molecules, was initially identified and characterized as a cellular interaction site for AGEs [6]. Growing evidence has indicated that *RAGE *is an important PRR, involved in amplification of pro-inflammatory response and antimicrobial host defense [7]. The present study, to our knowledge for the first time, investigated the potential clinical relevance of the genetic variants identified within the entire *RAGE *gene in the Han Chinese population. Three SNPs (rs1800625, rs1800624 and rs2070600) are selected from 10 SNPs with a minor allele frequency of more than or equal to 5% based on LD threshold and their possible functionalities. The 63 bp ins/del variant, although being rare in Western populations with an MAF of less than 1% [21] and not being included in the current public database of the Chinese Han population, is shown to be a common polymorphism in the Chinese Han population, showing 7.7% in Chongqing and 6.3% in Zhejiang populations, respectively, in our study cohorts. Although the rs2070600 polymorphism occurs in less than 5% in many populations, such as Brazilian and Finnish populations, the MAF was approximately 20% in Asia populations [50]. This is further confirmed in our study cohorts, showing an MAF of 29.4% and 27.4% in the Chongqing and Zhejiang districts. Therefore, the four genetic variants selected in this study cohort are all common gene polymorphisms in the Chinese Han population.

Case-control study is a common and convenient association study design for finding a genetic basis of disease. However, a major limitation in this approach is the potential for population stratification when inappropriate patient-control matching occurs, such as using healthy blood donors as the control group in the study. To avoid confounding association, we only selected trauma patients and prospectively followed them to determine whether those who had genetic variants had a lesser or higher risk of post-traumatic MODS and sepsis. In addition, the patients recruited in this study cohort were all Han Chinese. In the context of a biologically relevant phenotype and a racially uniform population, this might maximize the likelihood of finding a meaningful genetic association. Furthermore, two independent patient populations from different geographic regions were used in this study cohort. Our results indicate that among the four genetic variants, only the rs1800625 polymorphism reveals a strong clinical relevance, showing lower sepsis morbidity rate and MOD scores in the patients with the variant C allele both in Chongqing and Zhejiang cohorts. This is in contrast to its association with other diseases, showing increased risk of diabetes [18], diabetic retinopathy [19] and colorectal cancer [20]. This might be because *RAGE *plays different roles in the pathogenesis of different diseases. The other three genetic variants (rs1800624, rs2070600 and 63bp ins/del), being associated with an increased risk of some inflammatory diseases [18,26-28], are not shown to be associated with risk of sepsis and MODS in patients with major trauma in the Chongqing and Zhejiang cohorts. In combination with previous reports showing that the rs1800625 polymorphism is a common allele in other ethnic populations with the minor allele frequency of more than 10% [51-53], it might be used as a relevant risk estimate for sepsis and MODS in critical ill patients.

Given the functional significance of the rs1800625 polymorphism, we further investigated the association of this SNP with LPS-induced responsiveness of peripheral leukocytes obtained from trauma patients. The whole blood samples were taken immediately after admission in an attempt to avoid the potential effects of blood transfusion and fluid resuscitation. As we expected, the rs1800625 polymorphism is significantly associated with lower responsiveness of peripheral blood leukocytes in response to LPS stimulation, showing much lower levels of TNFα in patients carrying the variant C allele. This is in accordance with the clinical association of the rs1800625 with risk of sepsis and MODS in patients with major trauma. The rs1800625 polymorphism is located in the promoter region of the *RAGE *gene and induces T→C substitution at position -429 [54]. Bioinformatics analysis reveals that binding sites for some transcription factors (GATA-1, GATA-2 and sp1) (motif.genome.jp/) are present in close proximity to nucleotide -429 in the promoter. In order to further determine the functionality of the rs1800625 polymorphism, we investigated the effect of the rs1800625 polymorphism on the *RAGE *promoter activity using reporter gene assay system. Using the U937 cell line, we show that the fold increase of RLA is significantly lower in the cells transfected with vectors containing -429 C allele. It suggests that T→C variation at position -429 could significantly reduce the transcriptional activity of the *RAGE *promoter. Taken together, The T→ C variation at position -429 might reduce DNA-protein interaction, and then inhibit *RAGE *gene transcription, which contributes to decreased TNF production by peripheral blood leukocytes in response to *ex vivo *LPS stimulation, leading to decreased risk of sepsis and MODS in patients with major trauma.

There are some limitations to our current study. First, the sample size of both patient groups recruited in this study was relatively small, especially in the Zhejiang cohort. The values of power for rs1800625 in the Zhejiang cohort are shown to be 51.7% and 36.2% for sepsis morbidity rate and MOD scores, respectively, at a significance of 0.05. The too small number size of patients with the rs1800625 TC genotype (n = 5) and their higher incidence of sepsis (40%) in the Zhejiang cohort might explain the discrepancy in sepsis morbidity rate between the Chongqing and Zhejiang cohorts. The clinical relevance of the rs1800625 polymorphism needs to be validated in a larger population. Second, the population we studied was only Han Chinese who live in the Chongqing and Zhejiang districts. Our results may not be generalized to other populations. Third, difficulties in obtaining additional blood samples did not allow us to determine plasma sRAGE levels, or RAGE expression on peripheral blood leukocytes; therefore, the *in vivo *association between the rs1800625 polymorphism and *RAGE *activities needs to be confirmed. Despite the limited power of the clinical association study, our results demonstrate that the rs1800625 polymorphism might be a causal risk allele for sepsis and MODS in patients with major trauma

## Conclusions

The present study investigated the clinical relevance of the genetic variations within the entire *RAGE *gene by means of construction of haplotype bins in patients with major trauma. We demonstrate that rs1800625 polymorphism is shown to affect TNFα production, and might be used as a relevant risk estimate for sepsis and MODS in trauma patients. In addition, the rs1800625 polymorphism could significantly enhance the promoter activities of the *RAGE *gene.

## Key messages

• Among the four tagSNPs of *RAGE*, only rs1800625 reveals a strong clinical relevance, showing lower sepsis morbidity rate and MOD scores in the patients with the variant C allele in both the Chongqing and Zhejiang cohorts.

• The rs1800625 polymorphism is significantly associated with lower responsiveness of peripheral blood leukocytes in response to LPS stimulation, showing much lower levels of TNFα in patients carrying the variant C allele.

• The rs1800625 polymorphism could significantly enhance the promoter activities of the *RAGE *gene.

## Abbreviations

AGEs, advanced glycation end products; CHB, Chinese Han Beijing; DAMPs, damage-associated molecular patterns; DNA, deoxyribonucleic acid; ELISA, enzyme-linked immunoabsorbent assay; htSNPs, haplotype tagging single nucleotide polymorphisms; HWE, Hardy-Weinberg equilibrium; ISS, Injury Severity Score; LD, linkage disequilibrium; LP, length polymorphism; LPS, lipopolysaccharide; MAF, minor allele frequency; MHC, major histocompatibility complex; MODS, multiple organ dysfunction syndrome; OR, odds ratios; PAMPs, pathogen-associated molecular patterns; PCR, polymerase chain reaction; PRR, pattern-recognition receptor; RAGE, receptor for advanced glycation end products; RLA, relative luciferase activity; RPMI, Roswell Park Memorial Institute; SNPs, single nucleotide polymorphisms; TNFα, tumor necrosis factor alpha

## Competing interests

The authors declare that they have no competing interests.

## Author's contributions

LZ and A-QZ were the main researchers for this study and co-contributed to the writing of this manuscript. WG, JZ, L-YZ, D-YD, MZ, JY and CY were involved in the collecting of blood samples and other clinical data. H-YW did the technical work. J-XJ planned the study, wrote the protocol and was involved in the genetic and clinical aspects of data analyses and revised the manuscript. All authors read and approved the final manuscript.

## Supplementary Material

Additional file 1 **The definition of sepsis and infection**. We evaluated sepsis and infection of major trauma patients' according to these criteria. Click here for file

Additional file 2 **Table S1. Primers of the four variants of the *RAGE *gene and their PCR conditions**. The PCR primers, sequencing primers and the annealing temperatures of the four variants of the *RAGE *gene were shown in Table S1. Click here for file

Additional file 3 **The methods of plasmid construction**. The possible effect of -429T/C on the promoter activity was investigated using a reporter gene assay system. Two plasmids which contained the -429T promoter and -429C promoter were constructed. Click here for file
