# Supplementary material for: Identification of haplotype tag single nucleotide polymorphisms within the receptor for advanced glycation end products gene and their clinical relevance in patients with major trauma
Source: Crit Care. 2012 Jul 24;16(4):R131. doi: 10.1186/cc11436 (PMC3580716; doi:10.1186/cc11436)
Supplement: Additional file 1 — The definition of sepsis and infection. We evaluated sepsis and infection of major trauma patients' according to these criteria. [file cc11436-S1.DOC]

**Additional file 1**

Sepsis was defined if patients fulfilled all the following criteria: clinical evidence of infection, body temperature greater than 38.5°C or less than 36.5°C, and leukocyte count greater than 10×10^9^/L or less than 4×10^9^/L. Infection was defined as a clinically obvious source or positive bacterial cultures. Pneumonia was diagnosed when a predominant organism was isolated from appropriately obtained sputum cultures in the setting of purulent sputum production and/or a new or changing pulmonary infiltrate on chest x-ray film. Bloodstream infections were diagnosed based on isolation of a predominant organism from blood cultures obtained under sterile conditions. Criteria for urinary tract infections included >10 white blood cells/high power field on microscopic examination or isolation of >10^5^ organisms/ml urine or >10^4^ organisms with symptoms. Criteria for catheter-related infections included isolation of >15 colony forming units from catheter tips cultured only in the setting of suspected infection. Wound infection was identified by drainage of purulent material from the wound.
