# Supplementary material for: Identification of haplotype tag single nucleotide polymorphisms within the receptor for advanced glycation end products gene and their clinical relevance in patients with major trauma
Source: Crit Care. 2012 Jul 24;16(4):R131. doi: 10.1186/cc11436 (PMC3580716; doi:10.1186/cc11436)
Supplement: Additional file 2 — Table S1. Primers of the four variants of the RAGE gene and their PCR conditions. The PCR primers, sequencing primers and the annealing temperatures of the four variants of the RAGE gene were shown in Table S1. [file cc11436-S2.DOC]

**Additional file 2**

**Table S1. Primers of the four variants of the RAGE gene and their PCR conditions**

| **SNP** | **Primers for PCR** | **Sequencing Primer** | **SNP sequence** | **Annealing temperature** |
| --- | --- | --- | --- | --- |
| rs1800625 | F: bio-TCTTTTTTCCCTGGGTTTAGTTGA  R: ATAGGGTTCAGGCCAGACTGTTGT | GAGAGAAACCTGTTTGG | AA/GCTTC | 60°C |
| 63bp ins/del | F: AAA ACA TGA GAA ACC CCA GA  R: CCC CGA TCC TAT TTA TTC CA | - | - | 57°C |
| rs1800624 | F: CCCAGCCTTGCCTTCATGAT  R: bio-AGGGTTCAGGCCAGACTGTTG | ATGATGCAGGCCCAA | T/ATGCACC | 60°C |
| rs2070600 | F: ATTTGGATCCCCGTCACTCT  R: *bio-GCCTGGCACCGGAAAATC | CGTGTCCTTCCCAAC | G/AGCTCCC | 60°C |

*biotin labeling
