# Supplementary material for: Identification of haplotype tag single nucleotide polymorphisms within the receptor for advanced glycation end products gene and their clinical relevance in patients with major trauma
Source: Crit Care. 2012 Jul 24;16(4):R131. doi: 10.1186/cc11436 (PMC3580716; doi:10.1186/cc11436)
Supplement: Additional file 3 — The methods of plasmid construction. The possible effect of -429T/C on the promoter activity was investigated using a reporter gene assay system. Two plasmids which contained the -429T promoter and -429C promoter were constructed. [file cc11436-S3.DOC]

**Additional file 3**

Genomic DNA was obtained from subject homozygous for the T allele at position -429. The primers were 5'- TCGACGCGTTCCCTGGGTTTAGTTGAGAATTTT-3' (forward) and 5'-CCGAAGCTT TTCCGGCAGCCATCCTG-3’ (reverse). *Mlu I* and *Hind III* restriction sites (underlined) were introduced through PCR. After being digested with restriction enzymes *Mlu I* and *Hind III*, the PCR products of a 531 bp sequence (-504 ~ +26) of the RAGE gene were directly inserted into a promoterless pGL3-Basic vector (Promega, Madison, WI) containing the firefly luciferase gene as a reporter. The resulting construct containing the -429T promoter was then used to generate the construct containing the -429C using QuikChange Site-Directed Mutagenesis kit (Invitrogen, Carlsbad, CA) according to standard protocol. The mutation primers are 5’-TACTCTTTGGGGTCTTTTTTTTACTAAA-3’ (forward) and 5'- TCTTTCACGAAGCTCCAAACAGGTTTCTCT-3' (reverse). All constructs used in this study were confirmed by restriction map and direct sequencing (Takara Biotech, Dalian, China).
